# Water therapies (hydrotherapy, balneotherapy or aqua therapy) for patients with cancer: a systematic review

**DOI:** 10.1007/s00432-022-03947-w

**Published:** 2022-02-16

**Authors:** Maren Reger, Sabine Kutschan, Maren Freuding, Thorsten Schmidt, Lena Josfeld, Jutta Huebner

**Affiliations:** 1 Klinik für Innere Medizin II, Hämatologie und Internistische Onkologie, Universitätsklinikum Jena, Am Klinikum 1, 07747 Jena, Germany; 2 Supportive Care and Sportsmedicine, Universitätsklinikum Schleswig-Holstein, Campus Kiel, Krebszentrum Nord CCC, Arnold-Heller-Straße 3, Kiel, Germany

**Keywords:** Water therapies, Cancer, Aquatic therapy, Hydrotherapy, Balneotherapy

## Abstract

**Background:**

Water therapies as hydrotherapy, balneotherapy or aqua therapy are often used in the relief of disease- and treatment-associated symptoms of cancer patients. Yet, a systematic review for the evidence of water therapy including all cancer entities has not been conducted to date.

**Purpose:**

Oncological patients often suffer from symptoms which in patients with other diseases are successfully treated with water therapy. We want to gather more information about the benefits and risks of water therapy for cancer patients.

**Method:**

In May 2020, a systematic search was conducted searching five electronic databases (Embase, Cochrane, PsychInfo, CINAHL and PubMed) to find studies concerning the use, effectiveness and potential harm of water therapy on cancer patients.

**Results:**

Of 3165 search results, 10 publications concerning 12 studies with 430 patients were included in this systematic review. The patients treated with water therapy were mainly diagnosed with breast cancer. The therapy concepts included aqua lymphatic therapy, aquatic exercises, foot bathes and whole-body bathes. Outcomes were state of lymphedema, quality of life, fatigue, BMI, vital parameters, anxiety and pain. The quality of the studies was assessed with the AMSTAR2-instrument, the SIGN-checklist and the IHE-Instruments. The studies had moderate quality and reported heterogeneous results. Some studies reported significantly improved quality of life, extent of lymphedema, neck and shoulder pain, fatigue and BMI while other studies did not find any changes concerning these endpoints.

**Conclusion:**

Due to the very heterogeneous results and methodical limitations of the included studies, a clear statement regarding the effectiveness of water therapy on cancer patients is not possible.

## Introduction

Water therapies exist since antiquity. The Greek believed that there is a special healing power in water. The Romans built public baths that became recreational and social centers of the cities—precursors of today’s health resorts. In the nineteenth century, Vincenz Prießnitz and Sebastian Kneipp in particular emphasized the (further) development of water therapy: Prießnitz attempted to “harden” his patients with ice-cold water using “shock methods”. Pastor Sebastian Kneipp, on the other hand, used gentler methods of hydrotherapy. Since the middle of the last century, the use of spas as well as water exercises and hot and cold water became very common in medical treatments for relieving pain. (Bahadorfar 2014) Due to the development of new analgesic methods, the popularity declined but is still an important part of the treatment of patients with chronic pain. Due to overlapping treatment concepts, a strict differentiation between several types of water therapy is difficult, however, the therapeutic focus varies. In this present work, three types of water therapies are distinguished as follows:

Hydrotherapy applies water in all states of aggregation. The most utilized attribute of water used primarily in hydrotherapy is temperature. Cold water induces a centralization of the circulating blood to ensure a sufficient perfusion of vitally important organs by temporary peripheral vasoconstriction followed by vasodilatation. This stimulates the perfusion and should help to alleviate lymphedema or to heal chronic wounds (Mooventhan and Nivethitha 2014). For this reason, cold water may have analgesic and antiphlogistic effects. Warm water dilates the blood vessels and helps to relieve spasming or to relax muscles. Besides, hydrotherapy is effective in reducing high blood pressure (Jacob and Volger 2009) and relieving chronic back pain (Sawant and Shinde 2019). Examples for the use of hydrotherapy are Kneipp water baths or saunas.

Balneotherapy is known as a form of physical treatment with special baths. It is often used for relieving chronic pain as a common symptom of several illnesses like rheumatoid arthritis or fibromyalgia (Nasermoaddeli and Kagamimori 2005). In this case, special water enriched with e.g. iodide or carbonic acid is used. Additionally, balneotherapy contains other bathing forms like mud or moor bathing and is often used in spas for relieving chronic pain or mental complaints.

Aquatic therapy uses water exercises mostly performed in groups with a therapist in a therapy pool. The spectrum of the exercise techniques is very widespread and may contain practices to improve stretching, body strength or movement. In this therapy concept, the hydrostatic pressure of the water and the buoyancy are used, which may be helpful for people with musculoskeletal pain, orthopedic complaints or neurological disorders.

A large proportion of oncological patients suffer from symptoms which in patients with other diseases as for example rheumatoid arthritis are traditionally treated with therapeutic concepts including water therapy (Al-Qubaeissy et al. 2013). So far, only few data are known on these treatments in cancer patients and there is some discussion on whether water therapies are beneficial for cancer patients or may even put them at risks (for example infections, deterioration of lymphedema). To aggregate existing evidence, we conducted a systematic review in which we critically examined the existing evidence on the benefits and potential harms of water therapy in the treatment of cancer patients.

## Method

### Criteria for including and excluding studies in the review

Inclusion and exclusion criteria are listed in Table 1 based on a PICO-model. Generally, all study types were included if they used any intervention containing water therapy (see above for definition) and reported on any of the following patient-relevant outcomes: body functions like range of motion or status of lymphedema, the presence of painful trigger points, physical and psychological wellbeing, body image and participation in daily life after treatment of adult cancer patients. Type of treatment, frequency and duration was extracted. All reported adverse effects which appeared during water therapy were included in the review. Because of the wide range of types and application fields of water therapy, all cancer entities were included. Any kind of comparison group was eligible for this review, including watch and wait, standard care, land-based exercise, instructed exercises or diets.

**Table 1:** Inclusion and exclusion criteria

PICO	Inclusion criteria	Exclusion criteria
Patient	Cancer patients (all entities and stages) Adult patients (age > 18)	Patients with precancerous conditions or carcinoma in situ Preclinical studies
Intervention	Every intervention with balneo- or hydrotherapy (baths, aquatic exercises etc.)	Cryotherapy, for example with ice cubes
Comparison	All possible control groups (active control, placebo, standard/guideline/usual care, wait list)	
Outcome	All patient-reported outcomes including psychological outcomes (for example quality of life)	No patient-centered outcomes, for example laboratory parameters
Others	Language: German and English Full publication in peer reviewed journal Studies published since 1995	Grey literature (conference articles, abstracts, letters, ongoing studies, unpublished literature…)

Since little high-quality evidence was expected, systematic reviews and randomized controlled trials were included as well as controlled trials like cohort studies and case–control studies, one-armed studies, and case series and reports. Included patients were characterized by type and stage of cancer, type of treatment (e.g. chemo-, radiotherapy, operation), age and sex.

Oldest publication date was limited to 1995. In case no systematic review would be found within this time frame which included all former studies, the search would be extended to the beginning of the databases. Criteria for rejecting studies were primary prevention, grey literature not published in peer reviewed journals as full article, other publication type than primary investigation/report (e.g. comments, letters, abstracts) and study population with more than 20% children (under the age of 18) or precancerous conditions if results of adult patients with cancer were not reported separately. Additionally, studies were excluded if they reported no patient-centered outcomes (laboratory parameters except PSA which was considered as surrogate parameter for tumor progression of prostate cancer). Language was restricted to English and German.

### Study selection

**Fig. 1:**
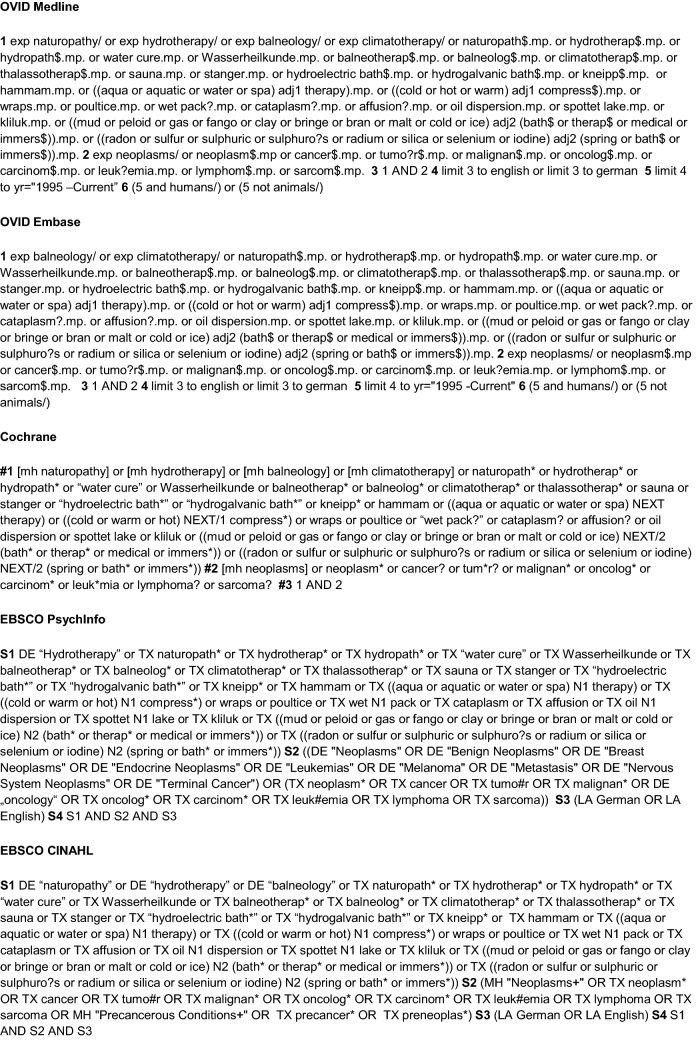
Search strings for different data bases

**Fig. 2:**
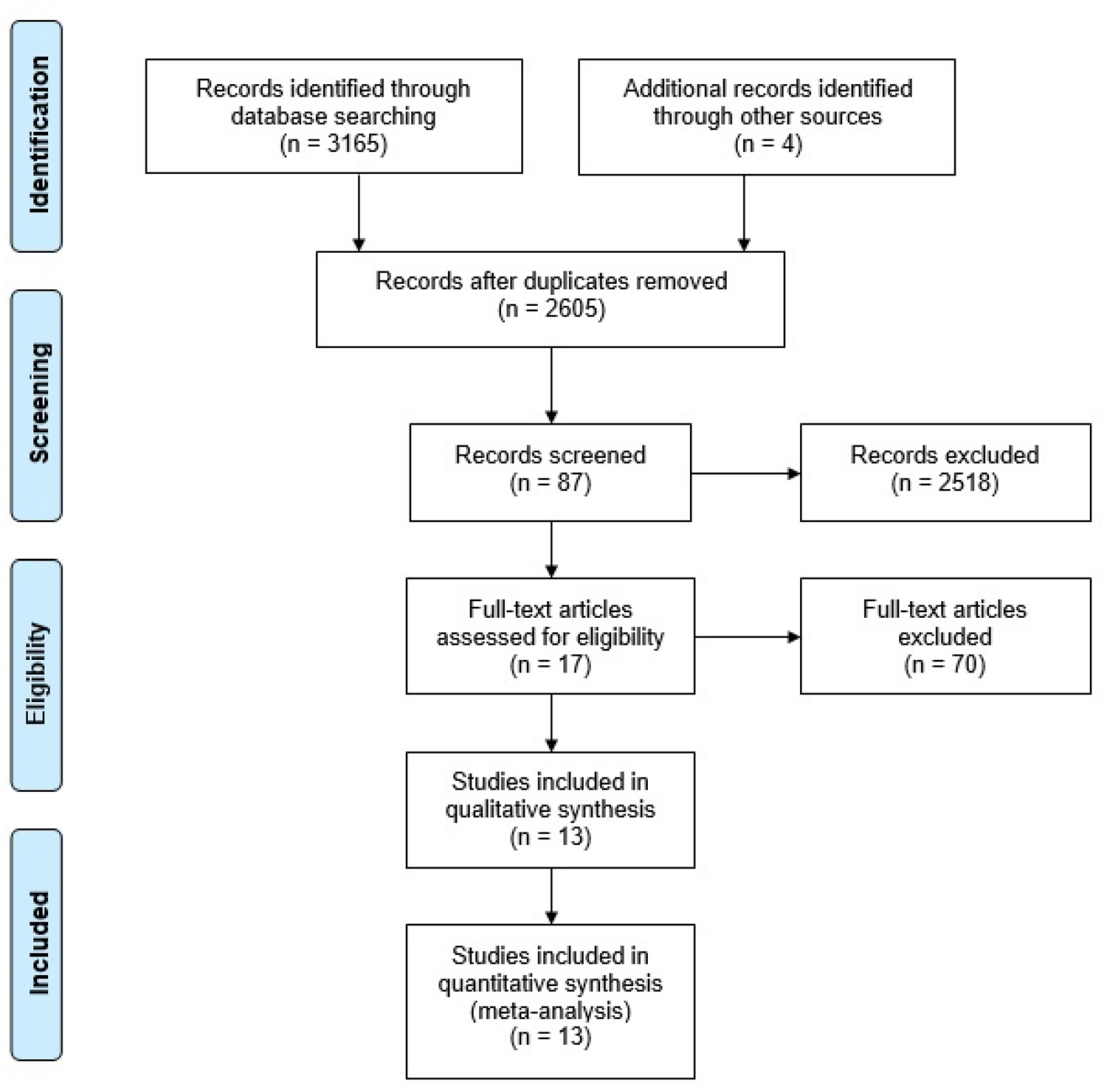
Prisma diagram (Moher and Tetzlaff 2009)

A systematic search was conducted using five databases (PubMed (Ovid), CINAHL (EBSCO), EMBASE (Ovid), Cochrane CENTRAL and PsycINFO (EBSCO)) in May 2020. Four additional studies were added by hand search. For each of these databases, a complex search strategy was developed consisting of a combination of MeSH terms, keywords and text words in different spellings connected to cancer and water therapy (Fig. 1). The search string was highly sensitive, since it was not restricted by filters of study or publication type. After importing the search results into EndNote X6, all duplicates were removed and a title and abstract screening was carried out by two independent reviewers (MR, JH). In case of disagreement, consensus was reached by discussion. After that, all full texts were retrieved and screened again independently by both reviewers. When title and abstract did not have sufficient information for screening purposes, a full-text copy was retrieved as well. Additionally, bibliography lists of all retrieved articles were searched for relevant studies. The flow of studies through the review can be seen in Fig. 2.

### Assessment of risk of bias and methodological quality

All characteristics of the included studies were assessed by two independent reviewers (MR, SK). In case of disagreement a third reviewer was consulted (JH) and consensus was reached by discussion.

### Methodological quality

The methodical quality of systematic reviews was assessed by the AMSTAR-2 instrument (Shea et al. 2017), (randomized) controlled studies by the SIGN-checklist 2 (Carolyn 2012), single-arm studies with the IHE-Instrument (Moga et al. 2012). The included studies were rated with the Oxford criteria (Phillips et al. 2009). In addition, blinding of researchers, blinding of outcome assessment and comparability of groups before treatment, not only in terms of demographic variables but also concerning the outcomes, was examined. Additional criteria concerning methodology were group size application of power analysis, dealing with missing data and drop-out (report of drop-out reasons, application of intention-to-treat-analysis), adequacy of statistical tests (e.g. control of premises or multiple testing) and selective outcome reporting (report of all assessed outcomes with specification of statistical data as the *p* value).

### Data extraction

Data extraction was performed by one reviewer (MR) and controlled by two independent reviewers (SK, JH). As a template for data extraction, the template of the German Guideline Program in Oncology was used. Concerning systematic reviews, only data from primary literature, meeting the inclusion criteria of the present work, were extracted.

## Results

The systematic search revealed 3165 results. Four studies were added after searching all reference lists. At first, duplicates were removed leaving 2605 studies. After screening title and abstract, 87 studies remained for complete review. Finally, ten publications were analyzed in this review, including one systematic review, two randomized controlled trials, five controlled trials and two case reports. In the systematic review, four studies were included of which three were considered relevant due to the inclusion criteria of this review. Accordingly, the ten publications reported data from twelve relevant studies. Detailed characterization of the included studies may be seen in Tables 2 and 3. Excluded studies are listed in Table 4. We did not extend our search before 1995 as relevant controlled studies from before 1995 should have been found and included in the systematic review by Yeung et al.

**Table 2:** Evidence tables of the included studies

Reference	Endpoints	Outcomes
Yeung (2018)	1. Lymphedema measured by water displacement 2. Physical function: strength, ROM (range of motion) 3. Symptoms: pain, heaviness, narrowness 4. Quality of life	Tidhar (2010): *n* = 48 1. No long-time effect of ALT reported SMD (95% CI) 0.12 (− 0.48; 0.72) 4. QoL with ULL27: no significant difference between groups in all three qualities SMD (95% CI): physical QoL: − 0.34 (− 0.95; 0.26), emotional QoL: − 0.53 (− 1.14; 0.08), social QoL: − 0.48 (− 1.09; 0.13) Johanson (2013): *n* = 25 1. Comparison of both arms: slightly, but not significantly better results in arm A than in arm B after treatment SMD (95% CI) 0.04 (− 0.75; 0.83), *p* > 0.05 2. No significant differences between the groups after three month SMD (95% CI): abduction: − 0.06 (− 0.85; 0.73), external rotation: − 0.51 (− 1.32; 0.29), flexion: − 0.92 (− 1.76; − 0.08) Letellier (2014): *n* = 25 1. Comparison of both arms: no significant differences between both groups SMD (95% CI): 0.19 (− 0.74; 1.13) 2. No significant differences between groups Grip strength: SMD (95% CI): healthy UL (upper limb): − 0.29 (− 1.22; 0.65), affected UL: 0.01 (− 0.92; 0.94), UL physical function DASH: − 0.11 (− 1.04; 0.82) 3. Significantly improved pain PPI with MPQ: SMD (95% CI): 0.71 (− 0.25; 1.68), *p* = 0.04 4. No significant difference of QoL between the groups FACT-B: SMD (95% CI): − 0.61 (− 1.56; 0.35)
Yamamoto (2011)	1. Activity of autonomous NS with HRV- analysis: HF, LF/HF, R-R-Interval 2. Neuroimmunological parameter with Cortisol and IgA concentration in saliva 3. Comfort/relaxation with VAS/FS	1. No significant changes in the activity of the autonomous nerve system before and after the treatment in both groups HF: mean (SD): Arm A t1: 2.69 (1.28), t5: 2.96 (1.79), Arm B t1: 3.67 (2.42), t5: 3.18 (1.39) LF/HF: Arm A t1: 2.82 (1.86), t5: 1.65 (1.23), Arm B t1: 1.37 (0.38), t5: 1.45 (1.24) R–R Interval: Arm A t1: 690.47 (163.08), t5: 721.39 (206.98); Arm B t1: 732.21 (198.57), t5: 740.21 (206.99) 2. No significant differences between the groups Cortisol µg/dl: mean (SD): Arm A pretest: 0.44 (0.52), posttest 0.27 (0.21), *t* = − 1.352, *p* = 0.218, Arm B pretest: 0.83 (− 1.26), posttest: 0.57 (0.66), *t* = 7 0.22, *p* = 0.330 IgA µg/ml: Arm A pretest: 25.16 (15.83), posttest: 69.17 (53.80), *t* = − 2.927 *p* = 0.019, Arm B pretest: 28.06 (23.60), posttest: 124.53 (162.30), *t* = − 1.907, *p* = 0.093 3. Significant increase of comfort in Arm A, no change in Arm B VAS “comfort”: mean (SD): Arm A pretest: 5.8 (1.04), posttest: 8.09 (1.69), *t* = − 3.488, *p* = 0.008, Arm B pretest: 6.09 (1.81), posttest: 6.41 (1.26), *t* = − 0.664, *p* = 0.525 VAS “relaxation”: Arm A pretest: 6.72 (1.95), posttest: 8.46 (2.15), *t* = − 2.414, *p* = 0.042, Arm B pretest: 6.69 (1.55), posttest: 7.12 (1.64), *t* = − 0.894, *p* = 0.397 VAS “wakefulness”: Arm A pretest: 5.57 (3.07), posttest: 5.98 (3.15), *t* = − 0.295, *p* = 0.775, Arm B pretest: 5.36 (2.53), posttest: 4.64 (2.87), *t* = 0.722, *p* = 0.491 VAS “pain”: Arm A pretest: 4.11 (2.58), posttest: 1.78 (1.82), *t* = 2.347, *p* 0.47, Arm B pretest: 2.44 (2.30), posttest: 2.54 (2.54), *t* = − 0.245, *p* = 0.813 FS: Arm A pretest: 5.22 (0.97), posttest: 3.56 (1.67), *t* = 3.162, *p* = 0.013, Arm B pretest: 4.56 (1.59), posttest: 4.67 (1.73), *t* = − 0.316, *p* = 0.760
Cantarero-Villanueva (2013)	Primary endpoints 1. Pressure pain threshold assessed with an electronic algometer Secondary endpoints: 2. Cancer-associated fatigue assessed with Piper Fatigue Scale 3. BMI calculated with height (stadio- meter) and weight 4. Waist circumference assessed with measurement tape 5. Weight	1. Significant increase of pressure pain threshold in intervention group for some muscle groups PPT: group-by-time interaction (95% CI), cervical: − 105.23 (− 162.11; − 48.35), shoulder (affected side:) − 56.05 (− 110.25; − 1.84), shoulder (non affected side): − 48.82 (− 99.58; 2.33), hand (affected side): − 47.42 (− 94.52; − 0.31), hand (non affected side): − 77.86 (− 128.69; − 27.04), tibia (affected side): − 116.50 (− 198.46; − 34.54), tibia (non affected side): -104.61 (− 176.07; − 33.16), *p* < 0.05 2. No significant increase of fatigue Piper Fatigue Scale: group-by-time interaction (95% CI): behavioral/ severity: 0.42 (− 1.12; 1.97), affective/meaning: 0.64 (− 0.80; 2.08), sensory: 0.75 (− 0.51; 2.01), cognitive/mood: 0.95 (− 1.14; 3.04), total fatigue score: 1.08 (− 0.05; 2.16), *p* < 0.05 3. No significant increase of BMI Group-by-time interaction (95% CI): 0.23 (− 0.35; 0.81), *p* < 0.05 4. Significant decrease of waist circumference in intervention group Tape measurement: group-by-time interaction (95% CI): 3.70 (0.78; 6.62), *p* < 0.05 5. No significant difference of weight in intervention group Group-by-time interaction (95% CI): 0.61 (− 0.71; 1.93), *p* < 0.05
Yang (2010)	Primary endpoints 1. Fatigue assessed with BFI-Taiwan 2. Sleep quality assessed with VSH-Scale 3. Vital parameters: temperature, heart rate, blood pressure Measurement time points: 1, 2, 4, 7, 14 days after chemotherapy	1. With second session, significant improvement of fatigue in intervention group: Mean (SD) BFI-Taiwan: first session: Arm A: 41.0 (1.8), Arm B: 41.7 (1.9), *p* value 0.79; second session: Arm A: 33.7 (1.9) Arm B: 44.1 (2.0), *p* < 0.001; third session: Arm A: 32.0 (2.3) Arm B: 48.6 (2.1), *p* < 0.001; fourth session: Arm A: 25.8 (1.8), Arm B: 46.7 (2.1), *p* < 0.001 2. Worse sleep quality in control group VHS- Scale: mean (SD) At sessions of chemotherapy First session: Arm A: 805.5 (29.3), Arm B: 743.0 (26.4) *p* = 0.11; second session: Arm A: 894.6 (26.4), Arm B: 750.2 (26.7), *p* < 0.001; third session: Arm A: 894.5 (26.2), Arm B: 753.8 (25.5), *p* < 0.001, fourth session: Arm A: 944.9 (21.7), Arm B: 763.2 (35.6), *p* < 0.001 3. Significant change of vital parameters Mean (SD) before- 1 min after foot bath- 20 min after foot bath Temperature: 36.4 (0.4), 36.7 (0.4), 36.3 (0.4), *p* < 0.001; heart rate: 80.9 (14.0), 81.6 (13.4), 79.2 (13.7), *p* < 0.001; systolic BP: 111.6 (15.1), 109.0 (13.7), *p* < 0.001; diastolic BP: 70.9 (9.3), 68.6 (/11.4), *p* < 0.001
Park (2015)	Primary endpoints: 1. Skin temperature assessed with a non-contact thermometer 2. Grade of neurotoxicity assessed with NCICTC 3. Calcium and magnesium in plasma of venous blood 4. QoL Assessed with FACT-C and FACT/GOG- NTx	1. Significantly higher temperatures in Arm A Mean (SD): forehead: Arm A Baseline: 35.99 (0.28), 1. Session: 35.96 (0.49), 8. Session: 35.91 (0.35) Arm B Baseline: 35.95 (0.21), 1. Session: 36.04 (0.16), 8. Session: 35.75 (0.22) feet: Arm A Baseline: 35.48 (0.35), 1. Session: 36.28 (0.52), 8. Session: 36.48 (0.56) Arm B baseline: 35.55 (0.35), 1. Session: 35.64 (0.19), 8. Session: 35.69 (0.23) *p* < 0.05 2. Changes of neurotoxicity-sensory grade in percent Arm A 25% better, 75% no change Arm B 8.3% better, 83.4% no change, 8.3% worse 3. No significant differences after treatment FACT-C and FACT/GOG-NTx: mean (SD): Calcium: Arm A before treatment: 8.83 (0.49), after treatment: 8.80 (0.45), *p* = 0.099; Arm B before treatment: 8.65 (0.62), after treatment: 8.54 (0.45), *p* = 0.313 Magnesium: Arm A before treatment: 2.01 (0.20), after treatment: 2.08 (0.40), *p* = 0.628; Arm B before treatment: 2.08 (0.26), after treatment: 2.09 (0.23), *p* = 0.288 4. Increase of QoL in foot bath group, decrease in foot massage group FACT-G: mean (SD) Arm A before treatment: 62.75 (11.29), after treatment: 65.33 (12.96), *p* = 0.028; Arm B before treatment: 59.63 (12.47), after treatment: 53.33 (11.09), *p* = 0.042 FACT/GOG-NTx: Arm A before treatment: 26.79 (4.81), after treatment: 31.13 (5.57), *p* = 0.568; Arm B before treatment: 29.41 (7.82), after treatment: 26.38 (7.75), *p* = 0.191
Lindquist (2015)	Primary endpoints: 1. Limb volume by water displacement or tape measurement Secondary endpoints: 2. BMI calculated with weight and height 3. Range of motion with goniometry 4. Daily physical function by using a DASH- and HOOS questionnaire 5. Questionnaire developed for the study to assess sexuality, body image, physical function and symptoms	1. Significantly better reduction of lymphedema in Arm A Water displacement/tape measurements: MD Arm volume: 0.185, *p* = 0.029; leg volume: 0.0872, *p* = 1.000 2. Significant reduction of BMI in Arm A, but no significant group difference BMI: mean (95% CI) Arm A: − 0.3 (− 0.5; 0.0), *p* = 0.047, MD: 0.872, *p* = 1.000 3. Significant improvement of rotation of the shoulder in Arm B ROM: MD before and after treatment (95% CI): 6.7 (1.7; 11.6), *p* = 0.012 Apart from this, no significant differences between the groups MD Elevation shoulder: 0.010, *p* = 0.014; abduction shoulder: 0.316, *p* = 0.229; external rotation shoulder: 0.316, *p* = 0.541; hip flexion: 0.220, *p* = 1.000; knee flexion: 0.078, *p* = 1.000 4. Significantly higher DASH- scores at patients with arm lymphedema in Arm B DASH and HOOS: MD: DASH: 0.174, *p* = 0.303; HOOS: 0.120, *p* = 1.000 5. No significant differences in all groups before and after treatment Questionnaire: results before/after treatment Frequency of arm/leg swelling: Arm A: 6/22 before treatment, 6/22 after treatment, *p* = 0.031; Arm B: 5/17 before treatment, 5/17 after treatment, *p* = 0.180; Arm C: 6/17 before treatment, 6/17 after treatment, *p* = 0.453 General well-being: Arm A: before treatment 5/24, after treatment 5/24, *p* = 1.000; Arm B: before treatment 6/19, after treatment 5/19, *p* = 0.453; Arm C: before treatment 5/18, after treatment 5/18, *p* = 0.344 Physical health: Arm A: before treatment 5/22, after treatment 5/22, *p* = 1.000; Arm B: before treatment 5/18, after treatment 5/18, *p* = 0.581; Arm C: before treatment 4,5/16, after treatment 5/16, *p* = 1.000 Lack of confidence in the own body: Arm A: before treatment 2/15, after treatment 2/15, *p* = 1.000; Arm B: before treatment 2/19, after treatment 2/19, *p* = 0.289; Arm C: before treatment 2/18, after treatment 2/18, *p* = 0.289 Feeling of damaged body: Arm A: before treatment 2/15, after treatment 2/15, *p* = 1.000; Arm B: before treatment 2/19, after treatment 2/19, *p* = 0.227; Arm C: before treatment 2/18, after treatment 2/18, *p* = 0.727
Fujimoto (2017)	Primary endpoints: 1. Vital parameters: heart rate, blood pressure autonomous neural activity measured with EKG 2. State of anxiety of the patients assessed with STAI Data was collected 30 min before and after treatment	1. No significant changes in vital parameters Mean (SD): Temperature: before treatment: 36.7 (0.5), after treatment: 36.7 (0.4), *p* = 0.8531; systolic blood pressure: before treatment: 117.0 (23.4), after treatment: 113.3 (21.2), *p* = 0.1575; diastolic blood pressure: before treatment: 67.5 (12.7), after treatment: 68.2 (11.2), *p* = 0.6835; heart rate: before treatment: 83.8 (15.4), after treatment: 81.2 (14.9), *p* = 0.0701; sympathetic neural activity: before treatment: 0.2 (0.1), after treatment: 0.2 (0.1), *p* = 0.7253; parasympathetic neural activity: before treatment: 0.5 (0.2), after treatment: 0.6 (0.3), *p* = 0.1609; activity of autonomous nerve system: before treatment: 1.5 (0.6), after treatment: 1.6 (0.9), *p* = 0.6431 2. Significant reduction of state of anxiety caused by the bath STAI: mean (SD): Before treatment: 47.7 (6.9), after treatment: 30.6 (4.9), *p* < 0.0001
Cantarero-Villanueva (2012)	Prim. endpoints 1. Neck and shoulder pain by VAS Secondary endpoints 2. PTT (pressure pain threshold): minimal pressure where a sensation of pressure first changes to pain assessed with an electronic algometer 3. TrPs (myofascial trigger points) Considered as active when both the local and the referred pain reproduced any pain syndrome which is recognized as familiar Pain is evoked by digital compression	1. Significant more pain reduction in Arm A than B VAS: Am A: mean difference over time MD[A] = MT1–MT0, Arm B: mean difference over time MD[B] = MT1–MT0, group difference over time MD[AB] = MD[A]–MD[B], (95% CI) Neck pain:—MD [A] = − 28, MD [B] = 3, MD [AB] = − 31 (− 49; − 22) Shoulder/axillary pain: MD [A] = − 15, MD [B] = 5, MD [AB] = − 19 (− 40; − 4) 2. Significant effect for facet joints PTT: Am A: mean difference over time MD[A] = MT1–MT0, Arm B: mean difference over time MD[B] = MT1–MT0, group difference over time MD[AB] = MD[A]–MD[B], (95% CI) C5–C6 zygapophyseal joint: affected side: MD [A] = 18, MD [B] = − 9.7, MD [AB] = 27.7 (3.9; 50.4),non- affected side: MD [A] = 0.1, MD [B] = − 18.2, MD [AB] = 18.1 (6.1; 52.2) Deltoid muscle: affected side: MD [A] = 25.5, MD [B] = 19.0, MD [AB] = 6.5 (− 35.7; 48.9), non-affected side: MD [A] = 21, MD [B] = − 0.3, MD [AB] = 21.3 (-15.8; 58.4) Second metacarpal: affected side: MD [A] = 33.4, MD [B] = − 12.7, MD [AB] = 46.1 (5.8; 86.2), non-affected side: MD [A] = 12.9, MD [B] = 15.7, MD [AB] = − 2.8 (− 45.3; 39.6) Tibialis anterior muscle: affected side: MD [A] = − 1.8, MD [B] = 18.9, MD [AB] = -20.7 (− 75.7; 34.5), non-affected side: MD [A] = − 6.9, MD [B] = 14.2, MD [AB] = − 21.1 (− 78.5; 36.4) 3. Significant differences in localization of TRPs in Arm A: less TrPs in hydrotherapy group Trapezius: affected side: *p* = 0.011; non- affected side: *p* = 0.042); levator scapulae: affected side: *p* = 0.039, non-affected side: *p* = 0.048); scalenus: affected side: *p* = 0.014, non-affected side: *p* = 0.001); pectoralis major: affected side: *p* = 0.017, non-affected side: *p* = 0.021); infraspinatus: affected side: *p* = 0.041, non-affected side: *p* = 0.034); sternocleidomastoideus: affected side: *p* = 0.0737, non-affected side: *p* = 0.787)
Tidhar (2007)	Primary endpoints 1. Leg volume measured by volumeter: circumference measured at added points, added together to calculate limb volume	1. Improvement of lymphedema Additionally less movement restrictions and softening of the fibrosis groin area Ability to work enhanced More days on which the patient was able not to wear compression garments without experiencing swelling of involved limb
Tidhar (2004)	Primary endpoints: 1. Volume of lymphedema Circumference of arm measured with tape, volume calculated	1. After one session Patient 1: Mean reduction of lymphedema 13% (55.5 ml); patient 2: No swelling, mean reduction of lymphedema 32.9 ml, patient 3: mean reduction of lymphedema 22% (87 ml) After 14 month: Patient 1: reduction by 249 ml (before treatment 2482 ml, after treatment 2058 ml), 8% difference between both arms after treatment, before only 21%; patient 2: reduction by 116 ml (before treatment 2556 ml, after treatment 2552 ml) before treatment 1% difference between both arms, after treatment -5%, subjective report of increased strength and self- confidence after ALT Patient 3: reduction by 326 ml (before treatment 3548 ml, after treatment 3222 ml, 13% difference between both arms before treatment, after treatment 2%

*Arm A* intervention group, *Arm B/C* control groups, *ALT* Aquatic lymphatic therapy, Brief Fatigue Inventory, *ROM* Range of motion, *SMD* Standardized mean difference, *PPI* Present pain intensity, *ULL27* Upper limb lymphedema—questionnaire, *MPQ* McGill Pain Questionnaire, *QoL* Quality of Life, *FACT-B *Functional Assessment of Cancer Therapy in patients with Breast cancer, *FACT-G* Functional Assessment of Cancer Therapy—General, *VSH* Verran and Snyder-Halpern sleep scale, *FACT/GOG-NTX* Functional Assessment of Cancer Therapy/Gynecologic Oncology Group-Neurotoxicity, *BP* blood pressure, *CIPN* Chemotherapy- induced peripheral Neuropathy, *FOLFOX* folinic acid, leucovorin, fluorouracil, oxaliplatin, *NCICTC* National Cancer Institute Common Toxicity Criteria, *DASH* Disability of Arm, Shoulder and Hand-questionnaire, *HOOS* Hip Osteoarthritis Outcome Score Questionnaire, *STAI* State-Trait Anxiety Inventory, *NS* nervous system, *HRV* Heart frequency variability, *VAS* visual analogue scale, *FS* Face Scale, *HF* high frequency, *LF* Low frequency

**Table 3:** Methodological quality

References	Study type	Standardized rating of risk of bias	Additional comments on methodology	Evidence Level (Oxford)
Yeung (2018)	SR	AMSTAR: Positive: 4 Partial Positive: 4 Negative: 6	PRO: detailed search string; study report; meta-analysis of 2 studies; assessment of risk of bias with PEDro scale CONTRA**:** moderate quality of all included studies; small patient samples (*n* < 50); no long-term results; no homogeneous diagnosis of lymphedema; no statements on conflicts of interests and blinding	1b-
Included studies:				
Tidhar (2010)	RCT	PEDro Score: 7/10	CONTRA: baseline differences between groups: higher rates of chemo- and radiotherapy in control group, higher rate of mastectomy in intervention group	
Johanson (2013)	RCT	PEDro Score: 7/10	CONTRA: no information on allocation concealment	
Letellier (2014)	RCT	PEDro Score: 6/10	CONTRA: no information on allocation concealment; high drop-out rate (28%); baseline differences between groups: intervention group lived already longer with lymphedema, lymphedema appeared earlier after surgery in control group	
Yamamoto (2011)	RCT	SIGN Positive: 3 Uncertain: 5 Negative: 1 Overall quality: acceptable	PRO: active therapy concept for control group CONTRA: very small sample size (*n* = 18), very short reporting: no information on adverse effects and CoI, very short reporting of the results, no information on drop-outs	2b-
Cantarero-Villanueva (2012)	RCT	SIGN Positive: 6 Uncertain: 0 Negative:2 Overall quality: acceptable	PRO: power analysis was conducted CONTRA: very small sample size (*n* = 20); no comparable training concept for control group; no active surveillance of adherence of control group; no statistical consideration of possible moderators such as the time with the therapist; potential multiple testing; pain measurement is based on subjective ratings; no information on assessment of adverse events	2b-
Cantarero- Villanueva (2013)	CT	SIGN Positive: 3 Uncertain: 2 Negative: 3 Does not apply: 1 Overall quality: acceptable	PRO: power analysis was conducted, no differences between groups and baseline concerning demographic and medical aspects(except: 12 patients take analgesics, higher rate of unemployment in control group), high level of adherence (> 79%), less Drop-outs CONTRA: no active surveillance of adherence in control group: no comparable training concept for control group, small patient sample, only subjective measurement of pain, potential multiple testing	3b
Yang (2010)	CT	SIGN Positive: 2 Uncertain: 3 Negative: 3 Dows not apply: 1 Overall quality: low	PRO: power analysis was conducted CONTRA: no information on comparability of groups at baseline, drop-outs higher than expected (14%, not analyzed separately), surveillance of the participants with telephone call not adequate for assessing the compliance, no telephone call in control group: placebo effect because of the conversation possible, very short reporting: no information on COI, adverse effects or blinding	2b-
Park (2015)	CT	SIGN Positive: 4 Uncertain: 1 Negative: 3 Does not apply: 1 Overall quality: low	PRO: quasi-experimental due to alternating group allocation, power analysis was conducted, groups and baseline comparable concerning use of painkillers, laboratory values, QoL, general characteristics and vital parameters, Bonferroni adjustment conducted CONTRA: small patient sample and high number of drop-outs (16,6%), no active training concept for control group, similar principle (foot bath) for foot massage group: bias and placebo effects possible, not all endpoints are patient-centered	3b
Lindquist (2015)	CT	SIGN Positive: 3 Uncertain: 3 Negative: 3 Overall quality: low	PRO: structured training concept for water and land group (arm A and B), very similar, valid and reliable measurement methods, groups comparable at baseline (except participants in water group were younger) CONTRA: no blinding, some outcomes were not assessed and analyzed in the control group, no structured concept in control group (arm C), no active surveillance, high number of drop-outs (19%)	3b-
Fujimoto (2017)	Single-arm	IHE Positive: 11 Unclear: 4 Negative: 5	CONTRA: no power analysis, referred to geographic region, small sample size (*n* = 24), 16% drop-outs, no control group, endpoints not assessed for every patient	4

*AMSTAR* A Measurement Tool to Assess Systematic Reviews, *IHE* Institute of Health Economics-Quality Appraisal Checklist for Case Series Studies, *PEDro* Physiotherapy Evidence Database, *SIGN* Scottish Intercollegiate Guidelines Network Methodology, *Checklist 2* randomised controlled trials

**Table 4:** Excluded studies

Author	Year	Title	Type	Reason for exclusion
Bolderston, Amanda	2006	The prevention and management of acute skin reactions related to radiation therapy: a systematic review and practice guideline	SR	No balneo- and hydrotherapy
Hayes, Sandi C	2009	Exercise and secondary lymphoedema: safety, potential benefits and research related issues	RCT	Multimodal training program with water- and land-based aerobics, which was not evaluated separately
Mourgues, Charline	2014	Positive and cost-effectiveness effect of spa therapy on the resumption of occupational and non-occupational activities in women in breast cancer remission: a French multicentre randomised controlled trial	RCT	Multimodal therapies (physiotherapy, diet, nutrition plan, exercises) which were not evaluated separately
Dalenc, F	2018	Efficacy of a global supportive skin care programme with hydrotherapy after non-metastatic breast cancer treatment: A randomised, controlled study	RCT	Patients received different treatments before conducting the study (e.g. massages, make-up- workshops)
Cai, P	2018	A Chinese medicine warm compress (Wen Jing Zhi Tong Fang), combined with WHO 3-step analgesic ladder treatment for cancer pain relief	RCT	Patients received mixture of different herbals combined with aquatic therapy, which were not evaluated separately
Deacon, R	2019	Does the speed of aquatic therapy exercise alter arm volume in women with breast cancer related lymphoedema? A cross-over randomized controlled trial	RCT	Primary outcome is not effect of water therapy, but comparison of two different therapy concepts

### Characteristics of included studies

Concerning all relevant studies, 430 patients were included and 397 of them were assessed due to 33 drop outs. The age of patients ranged from 18 to 78 years. 378 participants were female and 52 male.

The studies were carried out in Spain (Cantarero-Villanueva et al. 2013, 2012) Sweden (Lindquist et al. 2015), USA (Johansson et al. 2013), Canada (Letellier et al. 2014), Taiwan (Yang et al. 2010), Korea (Park and Park 2015), Israel (Tidhar and Katz-Leurer 2010) and Japan (Fujimoto et al. 2017; Yamamoto and Nagata 2011). In two studies, (Tidhar et al. 2004, 2007), no information on country and period of intervention was given.

The main cancer types of the patients were breast cancer (four studies, *n* = 206 (52%), references: (Cantarero-Villanueva et al. 2013, 2012; Tidhar et al. 2004; Yeung and Semciw 2018) and gynecological cancer (three studies, *n* = 113 (28%), references: (Lindquist et al. 2015; Tidhar and Katz-Leurer 2010; Tidhar et al. 2004). Further cancer entities were colorectal cancer (one study, *n* = 40 (10%), reference: (Park and Park 2015) and mixed groups with e.g. bladder, prostate, lung or liver cancer (two studies, *n* = 38 (10%), references: (Fujimoto et al. 2017; Yamamoto and Nagata 2011).

The intervention most frequently used was aquatic exercises like aqua lymphatic therapy (ALT) [three studies: (Tidhar et al. 2004, 2007; Yeung and Semciw 2018)] including aerobic, motility movements and stretching exercises inside a deep water pool [three studies: (Cantarero-Villanueva et al. 2013, 2012; Lindquist et al. 2015)]. Further, foot baths [three studies: (Yang et al. 2010; Park and Park 2015; Yamamoto and Nagata 2011)] and whole-body bathing [one study: (Fujimoto et al. 2017)] were examined. The duration of the interventions ranged from eight weeks up to 3 months.

In nearly all studies, water therapy was used as a complement to the main cancer therapy to alleviate the disease- and therapy-associated morbidity of surgery and chemotherapy. Primary cancer treatments were surgery such as mastectomy, vulvectomy or lymphadenectomy (Cantarero-Villanueva et al. 2013; Lindquist et al. 2015; Tidhar et al. 2004, 2007; Yeung and Semciw 2018), chemotherapy or endocrine therapy (Cantarero-Villanueva et al. 2012; Yang et al. 2010; Park and Park 2015). In two studies (Fujimoto et al. 2017; Yamamoto and Nagata 2011), water therapy was used as a palliative concept to alleviate the symptoms of the incurable cancer illness.

In the majority of studies, the control group did not receive any special therapy but was treated as usual. In one study that examined the effect of aquatic therapy on women with lymphedema after breast cancer (Letellier et al. 2014), the control group received compression sleeves and was advised to do a daily workout. Another study (Park and Park 2015) used foot massage instead of foot baths. In a three-armed study (Lindquist et al. 2015) concerning water exercise, the active control group carried out a land-based training program and the passive control group received usual care.

Due to the different types of therapies in these studies, several main patient-relevant endpoints are reported. For ALT and hydrotherapy, the most examined outcomes were the extent of lymphedema (five studies), quality of life (QoL, three studies), pain, trigger points (three studies), physical function including strength and range of motion (three studies), presence of fatigue (two studies), BMI and body weight (two studies). Patients treated with footbath and whole body baths were examined for vital parameters like heart rate, temperature, blood pressure (two studies) and the state of anxiety (one study). More details also on measurement instruments can be seen in the attachment.

### Excluded studies

Excluded were one systematic review (Bolderston et al. 2006) with other therapy concepts than water therapy and five RCTs with multiple interventions. As the effects of the single parts of these interventions are not known and were not analyzed separately, it is not possible to estimate whether the reported effects are caused by the water therapy or by a different treatment. In Hayes et al. (2009), the patients received a multimodal training concept composed of water- and land-based exercises. In Mourgues et al. (2014), the patients were not only treated with water therapy, but with multiple therapies like diet, nutrition advice and physiotherapy. In Dalenc et al. (2018), the patients within a group received several different treatments before conducting the study, for example massages, make-up workshops or showers. In Cai (2018), the patients received a mixture of different herbs along with aquatic therapy. In Deacon (2019), the primary outcome was not the effectiveness of aquatic therapy in cancer patients, but the comparison of two different therapy concepts.

### Risk of bias in included studies

The results of the assessment of bias for the included publications are presented in Table 3. Overall, the included studies only have moderate quality. Most of the two-armed studies do not have an active treatment concept for the control group, but only the recommendation to continue usual care. For this reason, performance bias cannot be excluded. Besides, the control group in all studies was not observed regularly in all studies. Therefore, it is unknown whether these patients used any additional treatment by their own regimen. Moreover, only five studies are single-blinded. Blinding is very difficult in these study concepts, nevertheless its absence leads to a high performance and detection bias, concerning subjective outcomes as quality of life, pain, anxiety and fatigue due to placebo effects caused by time spent with the therapist or due to patients’ beliefs in the effectiveness of the intervention. Some of the studies do not report all results of the assessed endpoints (Lindquist et al. 2015; Fujimoto et al. 2017), give little information on statistical methods and results, drop-out numbers and reasons; accordingly, reporting bias is moderate to high. Risk of detection bias is high in several studies because of missing data, e.g. insufficiently answered questionnaires and missing power analysis.

## Efficacy of water therapies

### Objective outcomes

#### Extent of lymphedema—aquatic therapy

Regarding all included studies, five reported results of aquatic therapy on the extent of lymphedema in breast cancer patients: three RCTs (Tidhar and Katz-Leurer 2010; Johansson et al. 2013; Letellier et al. 2014), which were also included in an systematic review (Yeung and Semciw 2018), one non-randomized prospective controlled study (Lindquist et al. 2015), two case reports on four patients (Tidhar et al. 2004, 2007).

The three RCTs applied water exercises with a temperature between 31 and 33.5° (Johansson et al. 2013; Letellier et al. 2014) and 28–29° (Tidhar and Katz-Leurer 2010) to the intervention group between 1 h/day for 8 weeks and half an hour per day for 12 weeks. The respective control group received no comparable treatment (encouragement to use compression garment, self-massage, and exercise was given to both groups and not supervised). Volume of lymphedema was assessed by water displacement. None of these studies found a significant difference between treatment and control arm after the treatment period. According to the PEDRO-Scale rating by the authors of the systematic review, all studies had a moderate risk of bias. Shortcomings in all cases were small sample sizes (*n* < 50), no information on dropouts, no active monitoring of the control group. In two cases, baseline data differed significantly between groups regarding rates of mastectomy chemo- and radiotherapy (Tidhar and Katz-Leurer 2010) and rates of time living with lymphedema and onset of lymphedema after surgery (Letellier et al. 2014).

The non-randomized prospective controlled study (Lindquist et al. 2015) compared aquatic therapy to a land-based training program and usual care. The volume of lymphedema was measured by water displacement or utilizing tapelines. Results showed significant reduction in volume of lymphedema in arms of participants after breast cancer therapy, but not in lymphedema of legs of participants who had gynecological cancer diseases (mean group difference, *p* value: arm volume: MD = 0.185, *p* = 0.029, leg volume: MD = 0.0872, *p* = 1.000). The authors admit that this difference could be caused by the low rate of cancer patients with leg lymphedema, so these results may be unreliable. Comparability of baseline characteristics in groups were checked, but there was a high drop-out rate of 19%. Due to a missing intention-to-treat analysis this leads to a high attrition bias. Furthermore, there is a high performance bias, as the land-based training program was not structured. Additionally, the study has a high reporting bias, as some outcomes were not assessed or evaluated for the control group.

Finally, four cases of breast cancer patients with lymphedema described in two publications by the same study group showed a reduction in volume of lymphedema assessed by a tapeline. The women were allowed to decide for themselves how often and which form of exercises they wanted to perform. Reporting was insufficient, especially in Tidhar et al. (2007), because of missing concrete data.

#### Effect on physical function—aquatic therapy

Three studies (Lindquist et al. 2015; Johansson et al. 2013; Letellier et al. 2014) reported data concerning the physical function of breast cancer patients with lymphedema neither of which showed any significant effects. The patients received aqua lymphatic therapy for 8 weeks (Johansson et al. 2013) respectively 12 weeks or 10 weeks (Lindquist et al. 2015).

In Johansson (Johansson et al. 2013), the control group received usual care, but no active training comparable to the intervention group. In Letellier et al. (2014), the patients were additionally encouraged to wear compression sleeves and the control group was scheduled to do a 30 min daily workout, instructed via DVD. Physical function, including strength and range of motion, was measured with hydraulic hand dynamometer and goniometry. In both studies, no significant difference between the two groups could be observed (Johansson et al. 2013): SMD (95% CI) abduction: − 0.06 (− 0.85; 0.73), external rotation: − 0.51 (− 1.32; 0.29), flexion: − 0.92 (− 1.76; − 0.08); Letellier et al. (2014): Grip strength: SMD (95% CI): healthy UL (upper limb): − 0.29 (− 1.22; 0.65), affected UL: 0.01 (− 0.92; 0.94), UL physical function DASH (Disabilities of Arm, Shoulder and Hand Questionnaire): − 0.11 (− 1.04; 0.82). Both studies were rated with a moderate risk of bias according to the PEDro scale. Allocation was not examined and in Letellier et al. (2014), a high rate of drop-outs (28%) led to a high attrition bias. In Lindquist et al. (2015), a three-armed study was conducted. Arm A received aquatic therapy once per week while arm B got a land-based training program and arm C continued usual care. The range of motion of several joints was measured with goniometry. No significant differences between the groups could be observed [MD (CI 95%) = healthy UL (upper limb): − 0.29 (− 1.22; 0.65), affected UL: 0.01 (− 0.92; 0.94)]. Risk of bias was moderate due to methodological shortcomings as explained above.

#### BMI/body weight: aquatic therapy

In two studies (Cantarero-Villanueva et al. 2012; Lindquist et al. 2015), the effects of water exercise on BMI and body weight in breast cancer patients with lymphedema were reported. In Cantarero-Villanueva et al. (2012), the intervention group received 24 sessions of aquatic therapy, but the control group was animated to continue usual care without a comparable training concept. Besides, there was no active supervision of the control group which leads to a high performance bias. Other shortcomings are the small sample size (*n* = 20) and missing randomization. No significant difference in BMI before and after treatment could be observed. Moreover, grip strength was not altered by the training SMD (95% CI): healthy UL (upper limb): − 0.29 (− 1.22; 0.65), affected UL: 0.01 (− 0.92; 0.94).

Lindquist’s three-armed study described above (Lindquist et al. 2015) reported a significant reduction of the BMI in the water exercise group [MD (CI 95%): *M* = − 0.3 (− 0.5;0.0), *p* = 0.047], but the statistical analysis did not show a significant group difference (mean group difference 0.872, *p* = 1.000). Due to the shortcomings mentioned above, the methodological quality of this study is limited.

#### Vital parameters-hydrotherapy/balneotherapy

Of all included studies, two Yang et al. (2010), Fujimoto et al. (2017) reported effects of water therapy on vital parameters in patients with mixed cancer entities. In both studies, the patients received baths to alleviate their symptoms fatigue, insomnia and anxiety.

In Yang et al. (2010), a two-armed study was conducted to estimate the effect of foot baths on gynecological cancer patients after therapy with platinum, while in Fujimoto et al. (2017), patients with palliative care received mechanical baths of the whole body.

In Yang et al. (2010), patients on chemotherapy in arm A received a 20-min foot bath daily, patients in arm B did not get any additional treatment. Changes in vital signs were considered significant (t1 = before treatment t2 = 1 min after treatment t3 = 20 min after treatment; body temperature: mean value (SD) t1 36.4 (0.4), t2 36.7 (0.4), t3 36.3 (0.4) *p* < 0.001, heart rate: t1 80.9 (14.0), t2 81.6 (13.4), t3 79.2 (13.7) *p* < 0.001, systolic blood pressure: t1 111.6 (15.1), t2 109.0 (13.7) *p* < 0.001, diastolic blood pressure: t1 70.9 (9.3), t2 68.6 (/11.4) *p* < 0.001). The study does not give any information on the comparability of both groups at baseline. Furthermore, there is a high attrition bias due to a high drop-out rate (14%) and lack of intention-to-treat analysis. The participants were only supervised via telephone calls, therefore it is questionable whether they were compliant or not. The participants in the control group did not receive any telephone calls. Therefore, an effect due to the conversation cannot be excluded. The reporting was insufficient concerning adverse effects, and the authors did not give a disclosure of conflicts of interest.

In Fujimoto et al. (2017), the patients received whole-body baths in half-seated or seated position. Additionally, they drank a glass of water before and after the treatment. Heart rate, blood pressure and sympathetic nervous system activity were measured by heart rate variability analysis 30 min before and after treatment. No significant changes in vital parameters were reported. The study had a very small sample size (*n* = 24) and a high drop-out rate which led to a high attrition bias. Furthermore, reporting was insufficient and endpoints were not assessed for every single patient. More information is listed in Table 3.

### Subjective outcomes

#### Quality of life—aquatic therapy, balneotherapy, hydrotherapy

Of all included studies, three examined the effect of water therapy on the quality of life in breast cancer patients with lymphedema. In Tidhar and Katz-Leurer (2010) and Letellier et al. (2014), the intervention group received aquatic therapy, whereas the control group continued their usual care. In Letellier et al. (2014), the patients additionally wore compression sleeves. In Park and Park (2015), the patients received hydrotherapy in the form of foot baths. The control group received foot massages and a 5-min foot bath before the massage.

Quality of life was primarily assessed with validated questionnaires. In Tidhar and Katz-Leurer (2010), the ULL27-questionnaire was used, whereas the FACT-B questionnaire was utilized in Letellier et al. (2014). Another version of the FACT questionnaire (FACT-C and FACT/GOG-NTx) was used in Park and Park (2015). While a significant improvement of QoL in the foot bath group compared to the foot massage group could be found in Park and Park (2015) (mean (SD): FACT-G: Arm A before treatment 62.75 (11.29), after treatment 65.33 (12.96), *p* = 0.042; Arm B before treatment 59.63 (12.47), after treatment 53.33 (11.09), *p* = 0,042 FACT/GOG-NTx: Arm A before treatment 26.79 (4.81), after treatment 31.13 (5.57) *p* = 0.568, Arm B before treatment 29.41 (7.82), after treatment 26.38 (7.75), *p* = 0.191), the other two studies did not show any significant differences between the groups.

However, there are several reasons why these results are insufficient to make a clear statement whether water therapy is useful for improving quality of life or not. First of all, not all patients answered the questionnaires completely. This lack of data may have caused a detection bias. Risk of performance bias cannot be ruled out either as the control group was a usual care group without data on what the patients actually did and whether they got any treatment at all that may have caused the reported differences between the groups. Another fact that restricts the significance of this study is the circumstance that the foot massage group received a 5 min-foot bath prior to treatment. Due to these very similar therapy concepts in both groups and the impossibility for blinding, placebo effects and detection bias have to be kept in mind.

#### Pain—aquatic therapy

Altogether, three studies (Cantarero-Villanueva et al. 2013, 2012; Letellier et al. 2014) reported the effects of water therapy on symptoms like pain in breast cancer patients with lymphedema.

In Letellier et al. (2014), pain is examined and collected by using a questionnaire (MPQ-McGill Pain questionnaire). The present pain intensity was measured and showed a significant decrease [inter-group difference (SMD, 95% CI) 0.71 (− 0.25, − 1.68), *p* = 0.04].

In Cantarero-Villanueva et al. (2013), one group (arm A) was treated with water exercise while the second group (arm B) continued on usual care. The primary endpoint was neck and shoulder pain reported by visual analogue scale (VAS). As secondary endpoints, the pressure pain threshold (PTT, minimal pressure at which the patient feels pain measured with an electronic algometer) and Trigger Points (myofascial trigger points measured by pressure stimulus) were reported. Significant differences between the groups could be found. The neck and shoulder pain was significantly lower in the aquatic therapy group ((median value (CI 95%), *p* value of group-by-time interaction) neck pain: − 31 (− 49, − 22) shoulder pain − 19 (− 40, − 4), *p* < 0.05). For the PTT, a significant effect could only be seen in the facet joints [median value (SD) affected joint 27.7 (3.9, 50.4) unaffected joint 18.1 (6.1, 52.2), *p* < 0.05]. In arm A, significantly fewer trigger points could be found for the trapezius, levator scapulae, pectoralis major, infraspinatus and sternocleidomastoideus.

In Cantarero-Villanueva et al. (2012), the intervention group (arm A) received 24 sessions of aquatic therapy, the control group (arm B) continued on usual care. The cut-off point of the pressure pain was measured as primary endpoint on several parts of the body with an electronic algometer. A significant increase of the pressure pain threshold in arm A was reported. The cervical and shoulder pain showed a greater decrease in the water therapy group compared with the control group. Results can be seen in Table 2.

#### Fatigue—aquatic therapy, balneotherapy

Two studies (Cantarero-Villanueva et al. 2012; Yang et al. 2010) measured the effects of water therapy on cancer-associated fatigue in patients with breast cancer and gynecological cancer. The results detected in these studies are contradictory.

In Cantarero-Villanueva et al. (2012), the intervention group (arm A) was treated with 20 sessions of aquatic therapy three times a week for 2 months, while the control group (arm B) received usual care. The cancer-associated fatigue was measured with the Piper fatigue scale, a validated numerical tool assessing subjective fatigue in four dimensions which are behavioral/severity, affective meaning, sensory, and cognitive/mood. In none of these dimensions, a significant difference could be seen. The detailed results are described in Table 2.

In Yang et al. (2010), gynecological cancer patients under platinum chemotherapy took a 20-min foot bath daily (hydrotherapy) one hour before going to bed, whereas patients in the control group did not. Compliance was monitored with a daily telephone call. Fatigue was measured by using the Brief Fatigue Inventory-Taiwan Form in four sessions. Higher scores indicate higher levels of fatigue. After the second session, a significant improvement of the fatigue in the experimental group could be observed [mean value (SD) first session: Arm A 41.0 (1.8), arm B 44.1 (2.0) *p* < 0.05, second session: Arm A 33.7 (1.9) Arm B 44.1 (2.0), *p* < 0.001, third session: Arm A 32.0 (2.3) Arm B 48.6 (2.1), *p* < 0.005, fourth session: Arm A 25.8 (1.8), Arm B 46.7 (2.1), *p* < 0.001]. As mentioned above, there was no information on the comparability of both groups at baseline and a high attrition bias due to high drop-out rates (14%) which were not analyzed separately.

#### State of anxiety—balneotherapy/hydrotherapy

In Fujimoto et al. (2017), 24 patients with terminal state of cancer disease with mixed entities received whole-body baths in half-seated or seated position and drank a glass of water before and after the treatment to prevent dehydration. Due to ethical considerations, it was only possible to conduct a one-arm study. The state of anxiety was measured with the State-Trait Anxiety Inventory 30 min before and after bathing. After the bath, a significant decrease of state of anxiety was reported [mean value (SD) before treatment 47.7 (6.9), after treatment 30.6 (4.9), *p* < 0.0001]. Endpoints were not assessed for every single patient and the small sample size (*n* = 24) with a 16% drop-out causes an attrition bias.

### Adverse effects

Only few studies report adverse effects or describe the methods to collect these data. In three studies (Johansson et al. 2013; Letellier et al. 2014; Tidhar and Katz-Leurer 2010), in which patients carried out water exercises, no adverse effects could be seen. In Tidhar and Katz-Leurer (2010), adverse effects were only reported as an increase of extremity volume measured by water displacement or an infection. In Johansson et al. (2013) and Letellier et al. (2014), no statement regarding the method of acquisition was made. One study examining the effect of aquatic therapy in breast cancer patients (Cantarero-Villanueva et al. 2013), found three patients with short-term edema and four patients with fatigue after the treatment, which disappeared after several days. In Cantarero-Villanueva et al. (2012), four cases of increased pain three days after treatment were reported. In Park and Park (2015), there were three patients with high fever and leukocytopenia and one case with nausea reported in the foot bath group. In the foot massage group, three cases with high fever and leukocytopenia could be observed. The publication does not report whether this problem was attributed to the intervention. These studies did not give any information on the method of assessment of the adverse effects either. In Fujimoto et al. (2017), the adverse events were collected by using a questionnaire. In this study, three cases with fatigue and exhaustion were reported. In the other studies, no information on adverse effects was given.

In general, water therapy has few side effects and can be safely used in cancer patients. Possible side effects are allergic reactions to bath additives used in balneotherapy, cardiovascular complaints due to vasodilation or fatigue. For this reason, contraindications are inflammations, severe cardiac diseases or vascular disorders.

## Discussion

The studies regarding the efficacy of water therapy on cancer patients were found to be very heterogeneous concerning the types of water therapy, type of cancer, reported endpoints and measurement methods to assess the outcomes.

Concerning the extent of lymphedema, positive effects of aquatic therapy have been reported only in the case reports and in one non-randomized controlled study, which had several methodological shortcomings and a high risk of bias. However, the three RCTs showed no significant differences due to aquatic therapy. All three RCTs lacked a power analysis and only included few patients (*n* < 50), thus rendering statistical tests more conservative and increasing the beta error (false negative) (Button et al. 2013). Moreover, there was no blinding and no active control or sham in the control groups. Thus, effects may be rather over- than underestimated. Moreover, two of the three RCTs (Johansson et al. 2013; Tidhar and Katz-Leurer 2010) lack comparability of the intervention and the control group concerning demographic characteristics at baseline, which makes results even harder to interpret. In summary, we have to state, that there is no reliable evidence that aquatic therapy affects the extent of lymphedema in general. In comparison to our systematic review, another study showed significant improvement in limb volume and range of motion. (Moher and Tetzlaff 2009) On the other hand, in contrast to widespread concerns of exercise in warm water increasing lymphedema, no increase has been shown in the studies.

All three studies (Cantarero-Villanueva et al. 2013, 2012; Letellier et al. 2014) examining the effect of water therapy on symptoms like pain or heaviness reported significant results. Nevertheless, there are several methodological limitations to the validity of these results. In Letellier et al. (2014), high drop-out rates (28%) led to a high attrition bias. Moreover, the assessment of pain by a questionnaire was very subjective. In Cantarero-Villanueva et al. (2013) and Cantarero-Villanueva et al. (2012), lack of blinding and comparable treatment concept for arm B as well as the control of adherence with questionnaires led to a high risk of performance and detection bias. Placebo effects may have been caused by other factors, for example time with the therapist. In Cantarero-Villanueva et al. (2012), there was a lack of controlling for multiple testing and a lot of single tests (15) were carried out. Besides, it is not clear whether water therapy could be helpful to increase muscular strength, because only grip strength was tested and no other muscle groups. This could be the reason why the authors found significant results for some muscle groups. Water therapy has proven effective on pain management in patients with fibromyalgia (Zamunér et al. 2019). Therefore, it can be assumed that water therapy could have positive effects on pain management in cancer patients as well.

Two studies (Cantarero-Villanueva et al. 2012; Yang et al. 2010) reported results concerning fatigue after water therapy. While in Cantarero-Villanueva et al. (2012) no significant differences between the groups could be observed, the level of fatigue was significantly lower in the treatment group than in the control group in Yang et al. (2010). This could be explained with the two different therapy concepts: while in Cantarero-Villanueva et al. (2012), the patients were treated with aquatic exercises, they received foot baths in Yang et al. (2010). Moreover, the significance of these results may be discussed. Firstly, data reports were very brief with no information on the comparability of the two groups before treatment, on blinding or concerning adverse effects. Moreover, the compliance of the participants was controlled by a telephone call and only in the intervention group which may lead to an effect due to the conversation. These methodological deficits could explain the different results in Yang et al. (2010) and Cantarero-Villanueva et al. (2012) concerning the effect of water therapy on fatigue. Other studies show that foot bathing is useful as supportive care to alleviate symptoms of the chemotherapy like CIPN (Mohamed et al. 2021).

Only one study (Fujimoto et al. 2017) examined the effect of whole-body baths on the state of anxiety in cancer patients. The results are considered significant, but it has to be taken into consideration that the study only treated patients with cancer in terminal state and therefore reflected only a small part of all cancer patients. Furthermore, it was only a small sample with high drop-out rates (16%) which leads to attrition bias. Regarding the actual ACSM guidelines, strong evidence of sport and physical activity on symptoms like pain, fatigue, lymphedema and physical function are reported. There are a lot of studies which prove that physical activity is helpful for cancer patients (Brown et al. 2014b). In our systematic review, the included studies do not reflect these results. This discrepancy might be due to a less intensive training in water therapy than in usual physical training. Campbell et al. (2019) recommend the FITT criteria consisting of frequency, intensity, time and type to implement a sufficient exercise load. The guidelines recommend a frequency of 2–3 times per week for 8–12 weeks at 60–80% RM (repetition maximum = amount of weight which can be lift for one repetition). The training should at least be conducted for 45–75 min and weights should be used, e.g. dumbbells or flexible bands. Large muscle groups should be trained following the principle “start low, progress slow”. This helps to reduce the extent of a lymphedema and the level of fatigue, and to reach a better quality of life. Regarding the included studies, the exercise concepts are very heterogenous and were not described in detail. Even though the majority of them (Cantarero-Villanueva et al. 2013, 2012; Lindquist et al. 2015; Tidhar et al. 2004, 2007; Yeung and Semciw 2018) applies ALT in a sufficient frequency and time, the intensity and type were not described in detail. While water was used for natural resistance, most studies did not use any additional weights. The missing effect of physical exercises in water might be due to missing intensity (exercises were performed without using any weights) and type (principle “start low, progress slow” was not implemented). Moreover, it should be considered which muscle groups might be strengthened by water therapy. Assessing hand grip strength may not be the adequate method to measure, for example, an increase in strength of the upper shoulder girdle or the limbs.

In general, the study design of controlled studies could be improved. Concerning aquatic therapy, blinding is nearly impossible. Nevertheless, establishing an active control group who could perform land based exercises instead of water exercises would be possible. Moreover, it might be a good idea to supervise the control group more consequently by structured questionnaires to reduce bias.

## Limitations of this work

There are several limitations of this systematic review. First of all, we excluded studies concerning children or teenagers. Furthermore, only studies in English or German language were included. Besides, articles published before 1995 were excluded. Yet, relevant controlled studies from before 1995 should have been found and included in the systematic review by Yeung et al. (Yeung and Semciw 2018) who performed a thorough search without limits on date of publication. Since they discovered no older studies, we may assume that no relevant data were missed due to the limit set in our search.

## Conclusion

Until now, it is not possible to give clear advice concerning the efficacy of water therapy on people with cancer, its benefits and risks. Some studies with moderate level of evidence show low effects on quality of life and lymphedema in cancer patients with treatment-induced lymphedema. Yet, data are missing on safety, especially for water with higher temperature. Moreover, the beneficial effect was shown only for active training in water and not for passive water treatments. Balneo- and hydrotherapy may be an additional treatment concept for people with cancer to reduce some symptoms and the adverse effects of cancer therapy as lymphedema, limited physical function, fatigue or pain. Activating types of water therapy should be preferred for cancer patients. In all cases, the oncologist should be consulted about risks and contraindications—like open wounds, infections or extremely high/low blood pressure—before starting water therapies.

All in all, more high quality evidence is necessary to confirm a significant benefit of water therapies and to disclose more clearly which type of therapy and which training protocol might be adequate for which group of patients.

## Data Availability

Electronical databases (Embase, Cochrance, PsychInfo, CINAHL, PubMed).
